# Real-time monitoring of human blood-brain barrier disruption

**DOI:** 10.1371/journal.pone.0174072

**Published:** 2017-03-20

**Authors:** Vesa Kiviniemi, Vesa Korhonen, Jukka Kortelainen, Seppo Rytky, Tuija Keinänen, Timo Tuovinen, Matti Isokangas, Eila Sonkajärvi, Topi Siniluoto, Juha Nikkinen, Seppo Alahuhta, Osmo Tervonen, Taina Turpeenniemi-Hujanen, Teemu Myllylä, Outi Kuittinen, Juha Voipio

**Affiliations:** 1 Department of Diagnostic Radiology, Medical Research Center (MRC), Oulu University Hospital, Oulu, Finland; 2 Oulu Functional NeuroImaging Group, Research Unit of Medical Imaging, Physics and Technology, the Faculty of Medicine, University of Oulu, Oulu, Finland; 3 Physiological Signal Analysis Team, Center for Machine Vision and Signal Analysis, MRC Oulu, University of Oulu, Oulu, Finland; 4 Department of Clinical Neurophysiology, MRC, Oulu University Hospital, Oulu, Finland; 5 Department of Anaesthesiology, MRC, Oulu University Hospital, Oulu, Finland; 6 Department of Oncology, MRC, Oulu University Hospital, Oulu, Finland; 7 Health & Wellness Measurement Group, Optoelectronics and Measurement Techniques Unit, University of Oulu, Oulu, Finland; 8 Department of Biosciences, University of Helsinki, Helsinki, Finland; Cleveland Clinic, UNITED STATES

## Abstract

Chemotherapy aided by opening of the blood-brain barrier with intra-arterial infusion of hyperosmolar mannitol improves the outcome in primary central nervous system lymphoma. Proper opening of the blood-brain barrier is crucial for the treatment, yet there are no means available for its real-time monitoring. The intact blood-brain barrier maintains a mV-level electrical potential difference between blood and brain tissue, giving rise to a measurable electrical signal at the scalp. Therefore, we used direct-current electroencephalography (DC-EEG) to characterize the spatiotemporal behavior of scalp-recorded slow electrical signals during blood-brain barrier opening. Nine anesthetized patients receiving chemotherapy were monitored continuously during 47 blood-brain barrier openings induced by carotid or vertebral artery mannitol infusion. Left or right carotid artery mannitol infusion generated a strongly lateralized DC-EEG response that began with a 2 min negative shift of up to 2000 μV followed by a positive shift lasting up to 20 min above the infused carotid artery territory, whereas contralateral responses were of opposite polarity. Vertebral artery mannitol infusion gave rise to a minimally lateralized and more uniformly distributed slow negative response with a posterior-frontal gradient. Simultaneously performed near-infrared spectroscopy detected a multiphasic response beginning with mannitol-bolus induced dilution of blood and ending in a prolonged increase in the oxy/deoxyhemoglobin ratio. The pronounced DC-EEG shifts are readily accounted for by opening and sealing of the blood-brain barrier. These data show that DC-EEG is a promising real-time monitoring tool for blood-brain barrier disruption augmented drug delivery.

## Introduction

The blood-brain barrier (BBB) inhibits the penetrance of hydrophilic and polar drugs into brain tissue and hinders effective use of treatments like methotrexate chemotherapy in the otherwise drug sensitive primary central nervous system lymphoma (PCNSL). It was recently shown that PCNSL relapses within 5 years in all subjects and over half of the subjects within 2 years with BONN intra-thecal reservoir treatment combined with multi-drug intravenous treatment and has dismal prognosis in a few months [1]. However, numerous preclinical [2–10] and clinical [1,11–15] studies have shown that transiently disrupting the BBB with hyperosmolar intra-arterial mannitol infusion during chemotherapy holds much promise as a therapeutic intervention for PCNSL [16] and markedly increases survival [17]. Results obtained using our modified BBB disruption (BBBD) method combined with a high-dose treatment protocol indicate 40-50% survival even in relapsed PCNSL for additional 7 years, and 100% disease free survival for 3 years in first-line cases with the treatment starting with BBBD [14].

The most widely accepted view of the mechanism underlying hyperosmolar mannitol-induced BBBD accounts for the barrier breach by osmotic shrinkage of endothelial cells and consequent opening of tight junctions between the cells [16]. The degree of the transient BBBD is crucial for the treatment with a direct link to patient outcome [18,19]. If the BBB is excessively opened, vasogenic edema and subsequent infarction will threaten the patient. On the other hand, if mannitol fails to make the BBB permeable to chemotherapeutic drugs, they do not reach the PCNSL cells behind the intact barrier, and the disease will progress. Evidently, means for real-time monitoring of the degree of BBBD during the barrier breach would be highly beneficial. However, so far there have been no quantitative ways to assess the degree of BBBD during therapeutic interventions.

Very low frequency (VLF, 0.01 – 0.15 Hz) oscillations up to 1-2 mV in the electrical potential of mammalian brain tissue were observed for the first time in electro-cortical experiments on rabbits [20]. In the 1970’s, large-amplitude brain-potential shifts evoked by respiratory acidosis in animal experiments were suggested to originate from a potential difference across the BBB [21–23]. Comparable mV-level shifts are seen upon voluntary hyper- or hypoventilation in scalp direct-current electroencephalography (DC-EEG) in humans [24] and upon corresponding respiratory changes in mechanically ventilated cats, where an even larger shift is brought about by BBBD [25]. All available evidence points to the BBB acting as a nonneuronal signal generator of such mV-level slow shifts measured at scalp [26]. However, signals generated by the BBB may also be coupled to neuronal function, since VLF oscillations in the human DC-EEG are synchronized with faster cortical EEG oscillations and they are phase-locked with slow fluctuations in brain excitability [27–29], suggesting a link between VLF oscillations and the mechanisms of neurovascular coupling at the level of BBB [27].

In this study, we hypothesized that therapeutic BBBD induced by intra-arterial mannitol infusion could be monitored using scalp DC-EEG. To test the hypothesis, we measured DC-EEG during routine clinical treatment of PCNSL patients while they received chemotherapy augmented with BBBD that we perform 2 to 4 times per week. We also monitored the subjects with near-infrared spectroscopy (NIRS) in order to collect information on cerebral hemodynamic that is known to play a role in DC-EEG signal generation [30]. We report for the first time pronounced DC-EEG shifts generated by BBBD upon intra-arterial mannitol infusions in human subjects. We also report the possibility to localize and monitor the BBBD using topographic analysis of DC-EEG data.

## Materials and methods

In this study 47 consecutive BBBD treatments were monitored in 9 PCNSL patients (mean age ±SD = 55±16 years, range = 20-68, 5 females). Sixteen of the infusions were introduced into the right internal carotid artery, thirteen into the left internal carotid artery and eighteen into the dominant vertebral artery. Patients were recruited in the study during 2014 and a written informed consent was obtained from each patient prior to the procedure in addition to routine clinical BBBD information. The study was carried out in accordance with the Declaration of Helsinki and approved by the Ethical Committee of Northern Ostrobothnia Hospital District, Oulu University Hospital (number 5/2014).

### BBBD procedure

The BBBD treatment for PCNSL in Oulu University Hospital is based on the original procedure of Neuwelt and coworkers [17,31], and we have been developing it further in collaboration with Neuwelt’s group since 2007. On the 1st treatment day the patient is imaged with MRI or CT and tested with routine clinical laboratory tests. Rituximab chemotherapeutic is given on the 1st day for metabolic activation of the drug in the liver prior to the BBBD treatment. On days two and three the patient is treated with BBBD-enhanced chemotherapy under general anaesthesia.

Before anaesthesia induction intravenous phenobarbital and midazolam are given. Anaesthesia is induced and maintained using propofol. Two to three minutes prior to intra-arterial mannitol infusion anaesthesia is deepened up to EEG suppression level (entropy 0) with a 250 mg intravenous thiopental bolus together with benzodiazepine. Atropine is given to counteract strong vasovagal effects of BBBD. Muscle relaxants are not used since they could impede detection of clinical seizures caused by the infusions.

The BBBD treatment, adopted in 2007 from the pioneering Portland group led by Edward Neuwelt, is given to one of the internal carotid arteries or to the dominant vertebral artery [31]. After angiographic verification of the selected artery, a hyperosmolal 25% mannitol (Hospira, Inc., Lake Forest, IL) bolus is administered intra-arterially in 30 s at an infusion rate of 4-6 ml/s, followed by 10-minute intra-arterial infusions of first methotrexate and then carboplatin (infusion rate 0.2-0.4 ml/min). Etoposide and cyclophosphamide are given intravenously 5 – 10 min prior to mannitol.

Intravenous contrast enhanced cone beam computed X-ray tomography (cbCT) (120 – 150 ml of Visipaque 270 mg/ml) is routinely used in our BBBD protocol to rule out excessive BBB opening that may lead to vasogenic edema requiring reversing cortisol treatment to close the BBB. Visipaque was given during carboplatin infusion and cbCT was performed immediately after the BBBD procedure was completed. The DC-EEG cap was removed for better image quality and region of interest measurements were performed on all major arterial territories to quantify the BBB status from cbCT data after the procedure using NeaView clinical analysis tool (Neagen, Helsinki, Finland). The timing of the Visipaque application (>10 min after mannitol infusion) and the sensitivity of cbCT limit the use of the present cbCT data to the detection of prolonged or excessive BBB opening. Cortisol treatment was not needed in any of the 47 treatments included in the present study.

### DC-EEG and ECG data collection and analysis

The term DC-EEG refers to recording EEG without any high-pass filtering [26,32,33]. DC-EEG data were recorded with a 32-channel MRI-compatible BrainAmp system (Brain Products) using Ag/AgCl electrodes (impedances < 5 kΩ) [34] placed according to the international 10-10 system. ECG was measured simultaneously with the same instrument near cardiac apex para-sternally. Data were sampled at 5 kHz and low-pass filtered at 250 Hz. Signal quality was tested before the BBBD procedure was commenced.

**Fig 1:**
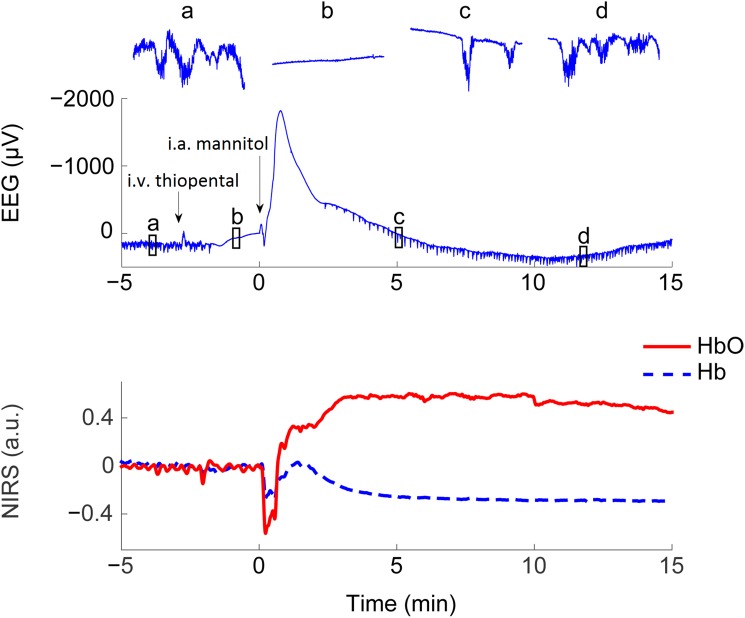
Characteristic EEG and NIRS responses seen during the BBBD procedure. Specimen traces illustrating simultaneous changes in raw EEG (upper graph) and NIRS signals (lower graph) during right carotid intra-arterial (i.a.) mannitol infusion. Deepening anesthesia with intravenous (i.v.) thiopental bolus (marked with an arrow) induces a baseline shift and suppresses activity at conventional EEG frequencies (insets a to d show 15 s sample traces on a 6 times expanded vertical scale) prior to mannitol infusion. Mannitol infusion (2nd arrow) then induces a multi-phasic potential response that begins with a pronounced negative shift reaching nearly -2000 μV in less than 1 min. The negative peak is followed by a slow potential descent below the pre-bolus level. Note the emerging burst-suppression (c) and subsequent faster EEG activity (d) similar to baseline state (a) as the thiopental effect slowly dissipates over 15 minutes. The simultaneously recorded NIRS graph shows first how the i.v. thiopental bolus produces a minor elevation to both NIRS HbO and Hb signals (red solid line and blue dashed line, respectively). When the 30 s i.a. mannitol infusion starts both NIRS signals plummet due to dilution of blood and they start to increase towards the original levels after the infusion. Subsequently, HbO rises above the baseline and stays there over the 15 minutes. On the other hand, Hb approaches the baseline level but soon starts to fall again obtaining a steady level clearly below the original baseline.

**Fig 2:**
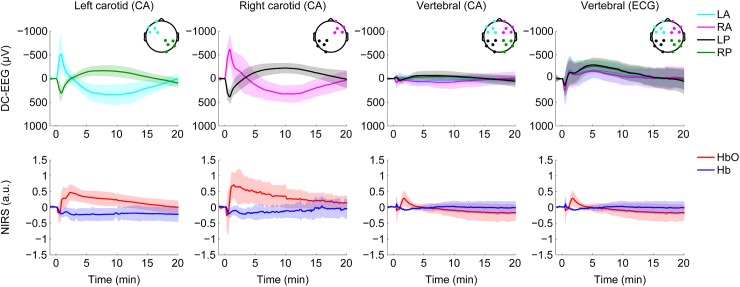
Grand average DC-EEG and average NIRS traces illustrating characteristic responses evoked by intra-arterial mannitol infusion. Each DC-EEG trace (upper panels; shaded area indicates values within ±1 SD) was generated by first calculating the mean of five electrode signals and then calculating the grand average of each recording. Mannitol infusion starts at time = 0 and lasts 30 s. DC-EEG responses upon carotid or vertebral artery mannitol infusion are shown using the common average montage (CA), however the responses to vertebral artery infusion are shown also after re-referencing to the distant ECG reference electrode (ECG; upper panel on the right). Bottom graphs show corresponding average oxy- and deoxyhemoglobin NIRS traces in arbitrary units (a.u.; ±1 SD). The number of NIRS recordings is less than that of DC-EEG, but all NIRS recordings are paralleled by simultaneously recorded DC-EEG data included in the upper graphs. Left (n = 13) and right (n = 16) carotid artery infusions induce a negative DC-EEG shift in electrodes above the treated arterial territory, which outlasts the infusion and is followed by a slower shift of opposite polarity. Contralateral posterior electrodes record a response that is qualitatively similar but reversed in polarity. A clear fall in the NIRS signals is seen during left (n = 8) and right (n = 8) carotid artery infusions, followed by a pronounced rise in HbO and a transient partial recovery of Hb after which Hb settles down on a level below the original baseline and HbO decreases slowly but does not fully recover. Vertebral artery infusions show a fronto-occipital DC-EEG potential shift (n = 18) without a lateralized effect, as expected. Re-referencing to the distant ECG reference electrode reveals that there is a negative shift throughout the entire scalp. The early transient shifts in the NIRS signals shown for vertebral artery infusions (n = 7) are more delayed and have much smaller amplitudes because NIRS was always measured on the forehead, i.e. they show responses generated in a non-infused brain area.

**Fig 3:**
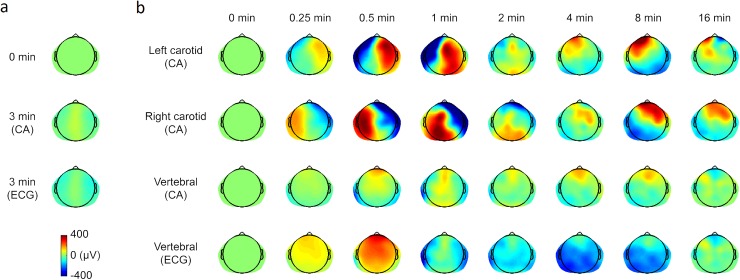
Spatiotemporal analysis of the DC-EEG data illustrated using heat maps. (a) Thiopental given at time 0 min generates a weak response in 3 min with slightly positive values along the midline and negative values at lateral electrode locations. Re-referencing the common average (CA) referenced data to the ECG reference renders the entire response slightly more negative. Data from all recordings (47 infusions) were pooled because thiopental was applied intravenously. (b) Temporal evolvement of the spatial distribution of DC-EEG responses to mannitol infusion shown using logarithmically increasing time intervals. The signal level preceding mannitol infusion (0 min) defines the zero level for the average responses calculated for 13 left carotid artery, 16 right carotid artery and 18 vertebral artery infusions. All data are shown using the CA reference montage. In addition, the bottom row of heat plots shows vertebral artery infusion data after re-referencing to the ECG reference.

Unless otherwise stated, EEG data were referenced to the common average and linear drifts were removed from all channels. This was done by subtracting off-line a linear trend from each individual channel after visual verification of the drift rate and its linearity throughout the entire recording period. Thereafter signals were downsampled to 1 Hz (anti-aliasing with a FIR filter) and low-pass filtered using a 21-point moving average filter for detection of infraslow EEG signals (i.e., DC shifts). The specimen trace (Fig 1) is shown using a wider bandwidth (channel F4, low-pass cut-off at 48 Hz). Average responses (Fig 2) were calculated for left anterior (Fp1, F3, F7, FC1, FC5), right anterior (Fp2, F4, F8, FC2, FC6), left posterior (P3, O1, P7, CP1, CP5), and right posterior (P4, O2, P8, CP2, CP6) channels. When re-referencing to the ECG reference was done, the ECG reference electrode signal was low-pass filtered like the DC-EEG channels. EEGLAB [35] was used for topographic illustrations (Fig 3) of DC-EEG data based on all the 31 recorded channels of the 10-10 system (Fp1, Fp2, F3, F4, C3, C4, P3, P4, O1, O2, F7, F8, T7, T8, P7, P8, Fz, Cz, Pz, Oz, FC1, FC2, CP1, CP2, FC5, FC6, CP5, CP6, TP9, TP10, POz).

### NIRS data collection and analysis

Each subject was measured with one NIRS channel placed on the forehead beneath the EEG-cap lead adjacent to Fp1 or Fp2 leads on the side of the infused artery using a source-detector distance of 3 cm. NIRS data was recorded using a NIRS measurement device utilizing wavelengths of 660 nm and 830 nm [36]. The sampling rate of NIRS data acquisition was 1 kHz. Temporal changes of Hb and HbO concentrations were calculated from raw NIRS time courses using MATLAB’s NIRS processing package called HomER2 [37]. Hb and HbO data were then low pass filtered with the cut-off frequency at 0.15 Hz.

### Anaesthesia monitoring

Cardiovascular signals (ECG, SpO2, EtCO2, intra-arterial blood pressure) were collected simultaneously using GE Datex-Ohmeda S/5 Avance system for routine patient surveillance and for verification of BBBD-induced vasovagal changes (data not shown).

## Results

### Mannitol-induced BBBD generates pronounced DC-EEG shifts

Mannitol infusion induced a robust multiphasic response in EEG channels monitoring the affected arterial territory, with amplitudes that were orders of magnitude larger (up to 2 mV) than those of commonly observed EEG rhythms. In the middle of the infused carotid arterial territory (electrode F4 for right and F3 for left carotid artery infusion) the response to the 30 s mannitol infusion typically commenced with a negative peak lasting 1.5-2 minutes and coinciding with the first (and second) pass of the intra-arterial mannitol bolus (Fig 1). This initial response was paralleled by robust responses in the simultaneous NIRS measurement (see below) and followed by a prolonged (10-15 min) DC-EEG shift of opposite polarity.

The BBBD procedure includes an intravenous thiopental bolus 2 min prior to mannitol. Thiopental caused a negative baseline shift of about 100 μV at F4 by the time of the mannitol application, and it completely abolished neuronal activity seen at frequencies > 0.5 Hz (Fig 1). After about 4 minutes the thiopental effect faded and faster rhythmic activity with burst suppressions reappeared on the EEG. Importantly, the pronounced DC-EEG potential shifts brought about by mannitol occur despite the absence of rhythmic neuronal activity, which together with their high amplitude suggests their non-neuronal origin.

**Fig 4:**
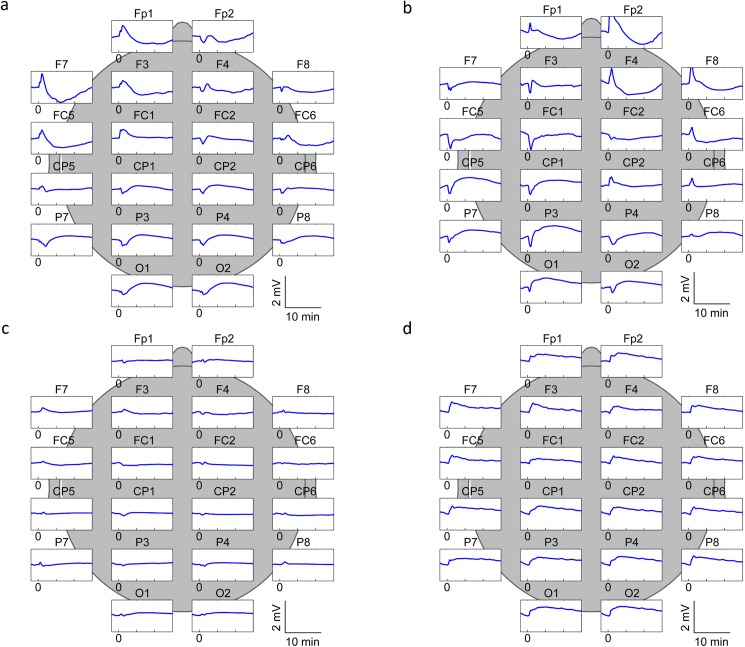
Typical spatial distribution of the DC-EEG responses. Specimen traces recorded during the BBBD procedure with left (a), right (b) and vertebral (c, d) artery infusion of mannitol. On average, carotid artery infusion evoked responses of the kind shown in Fig 1 in electrodes of the 10-10 system located anterior to vertex on the side of the infusion, whereas posterior electrodes on the opposite side showed rather similar responses with opposite polarity (common average reference montage). Therefore, the four subsets of five electrodes shown here were chosen for further characterization of the signals. When using the common average montage, responses evoked by vertebral artery mannitol infusion (c) were strikingly small in amplitude compared to those seen upon carotid artery infusion, suggesting in the former case the presence of a uniformly distributed signal component that cancels out in a differential recording against the common average reference. A reference point distant to the electrodes of the 10-10 system was provided by the ECG reference electrode, and indeed, re-referencing to the low-pass filtered ECG reference electrode signal revealed a prolonged negative shift (d).

The spatiotemporal characteristics of the DC-EEG shifts were analysed using more heavily low-pass filtered signals (see Materials and Methods). Typical spatial distributions of the DC-EEG potential shifts upon a left and a right carotid artery mannitol infusion are illustrated by a family of traces (Fig 4A and 4B respectively). Shifts qualitatively similar to those at F4 with right carotid artery infusion (or F3 with left infusion) were seen throughout the infused arterial territory, whereas contralateral (especially posterior) electrodes recorded a parallel sequence of shifts but with slightly lower amplitude and opposite polarity. Vertebral artery mannitol infusion resulted in responses with much lower amplitudes and a less salient spatial distribution (Fig 4C). Since a DC-EEG shift that is uniformly distributed throughout the scalp cannot be detected when using the common average montage, we re-referenced the vertebral artery infusion responses using the electrocardiogram (ECG) reference. This revealed a prolonged negative shift with no lateralization, and again, with highest amplitudes above the infused vertebral territory (Fig 4D).

Grand average DC-EEG traces at opposite quarters of the scalp were calculated to illustrate characteristic responses evoked by a total of 47 intra-arterial mannitol infusions (Fig 2). On average the negative peak seen at frontal sites on the side of carotid artery infusion had an amplitude of -560 μV (n = 29), and the slower positive shift peaked at 330 μV and lasted for 10 to 15 min. With vertebral artery infusion, the slow negative shift seen at posterior sites peaked at about 5 min and had an amplitude of -65 μV or -275 μV (n = 18) when using the common average reference or the ECG reference, respectively. In all cases, the responses are fading at the end of the 20 min monitoring of the mannitol effect.

A more detailed spatial mapping of the DC-EEG shifts is shown using topographic heat maps (Fig 3). The intravenous thiopental induces a rather uniformly distributed small shift by the time of mannitol infusion (Fig 3A) that is taken as the zero level for the average heat maps showing responses to mannitol (Fig 3B). The high amplitude and robust lateralization of responses is striking with carotid artery infusions, whereas vertebral artery infusion causes minimally lateralized responses with frontal-posterior differences. Re-referencing reveals that both the thiopental and vertebral artery mannitol responses are significantly attenuated when using the common average reference montage.

### The time courses of NIRS and DC-EEG responses differ

Both oxyhaemoglobin (HbO) and deoxyhaemoglobin (Hb) NIRS signals plummeted during the 30 s mannitol infusion (Figs 1 and 2). The drop was quickly followed by a hyperaemic increase in cortical oxygenation (30 s – 2 min) and an increase in HbO to a substantially elevated level. The increase in HbO persisted until the end of the 20 minute measurement period showing a slow and rather linear trend towards the original baseline level.

The Hb signal behaved differently compared to HbO. After the mannitol-bolus induced initial fall, Hb temporarily increased towards its original value. This was followed by a prolonged decrease below the original baseline and a very slow recovery towards the original pre-bolus level.

The NIRS measurements were performed always on the forehead due to imaging- and procedure-related limitations during the BBBD. The results were concordant between both carotid artery BBB disruptions, and the very prolonged cerebrovascular response was strikingly different compared to the gradually fading DC-EEG shifts. Obviously, a frontal NIRS cannot monitor the vertebral artery territory, and therefore the much less prominent NIRS responses evoked by vertebral artery mannitol infusion are shown only to illustrate the effects of diluted mannitol in a non-infused cortical area.

### cbCT results rule out excessive BBB opening

**Fig 5:**
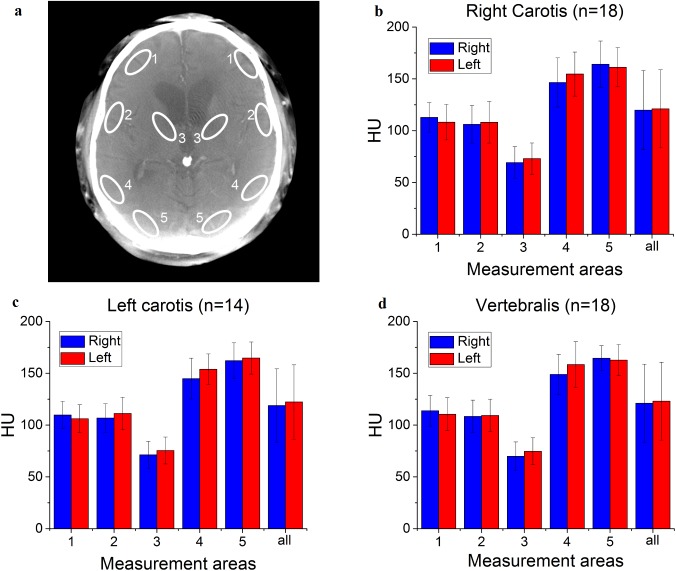
Region of interest analysis of the cbCT following i.a. multi-chemotherapy. Selected major territories are described using white ellipses marked by numbers 1-5 bilaterally (a). HU-values calculated from these areas and mean of them (named as ‘all’) are illustrated by treated artery; right carotis (b), left carotis (c) and vertebralis (d). In every case right (blue) and left (red) side are separated and also SD bars are represented.

We estimated whether excessive BBB opening occurred by comparing the enhancement of grey matter in the treated vascular territory vs. the non-treated arterial territories from cbCT data (Fig 5). The results indicated that none of the treated vascular territories showed increased brain tissue enhancement, i.e. the BBB did not leak iodine-contrast molecule complexes into the brain tissue in excess amounts ruling out excess or prolonged BBBD requiring cortisol treatment. Taking into account the limitations of the present cbCT method (see Materials and Methods), this result is in good agreement with our DC-EEG results which suggested that BBB closes by the end of the BBBD treatment.

## Discussion

The results of this study demonstrate for the first time real-time monitoring of human therapeutic BBBD using scalp-recorded DC-EEG. BBBD was induced with intra-arterial mannitol infusion in anesthetized patients receiving chemotherapy for PCNSL. DC-EEG detected robust mV-level shifts providing spatiotemporal information on the course of the induced BBBD. Simultaneously measured NIRS detected dilution of blood upon intra-arterial mannitol infusion followed by marked alteration in oxygen extraction fraction. Since the intact BBB makes the brain parenchyma inaccessible to hydrophilic drugs, new diagnostic and therapeutic innovations involving BBBD may benefit from the methods described here.

The BBB forms the first line defence of the brain against various forms of pathogens present in blood, and it also prevents targeting the brain parenchyma with systemic administration of hydrophilic medications. The interest in the role of BBB in brain pathology and in treating brain diseases has increased over the past few years [38,39]. PCNSL is one of the most aggressive brain tumours and it is commonly treated using methotrexate [17,40]. Progression-free survival time counts in months and mortality is high, even with high-dose intravenous methotrexate or intra-thecal chemotherapy [17,40]. Tumour growth affects BBB integrity locally and makes systemic chemotherapy possible. However, the lymphoma cells form micro-metastases into areas behind intact BBB, which prevents the penetrance of hydrophilic agents into the otherwise chemosensitive malignant neoplasms. Therefore, BBBD has been combined with methotrexate in order to cure PCNSL [16,17,31]. To this end, intra-arterial hyperosmolar mannitol infusions are safely used to transiently permeabilize the BBB, which increases the brain penetration of macromolecule chemotherapeutics by up to 100-fold and thereby improves the response to treatment [17,31,39,41].

The ability to monitor the degree and duration of BBBD is crucial for the treatment of PCNSL as sub-optimal or excessive BBB opening increases mortality and complications [19]. Previously, there have been no real-time methods to monitor BBBD. Computed tomography (CT) has been used to give information on the state of the BBB [42,43] but it is inaccurate, and continuous methods are needed for optimization of the BBBD level with drugs. Our present data show that the readily applicable method of DC-EEG, preferably combined with NIRS, offers the possibility to monitor the level of BBBD in real time. In order to establish a quantitative relationship between DC-EEG responses and BBBD, future studies are needed where an independent measure of BBB opening is used in parallel with DC-EEG. In this respect, an intriguing opportunity is serum S100β, which has been used as a biomarker for BBB opening in a virtually identical study [15].

The tight BBB maintains a trans-endothelial voltage between blood and brain tissue [21,23,44]. This voltage is a consequence of unequal endothelial cell apical and basolateral membrane potentials, and comparable with the trans-epithelial potential differences that are observed in some other tissues. The human brain is positive with respect to blood by 1 to 5 mV [45], and changes in this potential can cause up to mV-level shifts in human scalp DC-EEG [24,30]. Assuming that BBBD shunts the positive voltage maintained by intact BBB, the predicted initial human DC-EEG response that indicates BBB leakage will be a negative shift, and re-sealing of the BBB will finally restore the original signal level. This is exactly what we observed in DC-EEG channels above the perfused arterial territory. However, even if mannitol did not induce a simple short-circuiting of the BBB (see [46]), DC-EEG responses can be used to monitor the spatiotemporal behavior of mannitol-induced effects on the BBB. Currently there is no information available on the course of human brain-blood voltage changes during prolonged permeabilization of the BBB in manoeuvres of the present kind. Obviously scalp DC-EEG signals will reflect varying diffusion potentials across the disrupted BBB that get mixed with signals generated simultaneously by the still intact parts of the BBB while they react to the circulating diluted mannitol bolus. It is also reasonable to assume that local leaks across the BBB associated with tumor growth may to some extent reduce the steady state BBB potential difference that then gets more robustly shunted by BBBD. Our results are in line with the above predictions and a previous study [25], where BBBD was induced with sodium dehydrocholate (DHC) or mannitol in anesthetized cats, resulting in millivolt level potential shifts in DC-EEG. The possibility that cortical spreading depression (SD) is involved in the BBBD-induced DC-EEG responses was excluded in the above-mentioned study on cats, and more evidence against BBBD triggering SDs comes from a study on anesthetized rats where carotid artery infusion of hypertonic mannitol was used to induce BBBD and SDs were triggered only much later upon prolonged infusion with a high K^+^-solution [47]. It is obvious that more work is needed in order to obtain more detailed mechanistic insights into how BBB generates DC-EEG signals upon mannitol infusion. An optimal preparation for future work might be the isolated whole brain preparation maintained *in vitro* by arterial perfusion [48,49].

Intra-arterial infusion of detergents like DHC can induce vascular thrombosis and be lethal in animal experiments, and some researchers suspect that other hyperosmolar solutions carry a potential for ischemia or cerebral haemorrhage [46]. In brain tumour patients the use of intra-arterial mannitol and chemotherapy has been shown to present some non-specific white matter lesions and two ischaemic lesions in fifteen patients that underwent 318 procedures [13]. However, all of these subjects maintained their level of cognitive and neurologic function and the magnetic resonance imaging (MRI) findings did not have a correlate in cognitive tests [13]. When compared to the short life expectancy in PCNSL, and complications following other treatments, the use of hyperosmolar intra-arterial mannitol for BBBD can be readily justified to be safe as it markedly prolongs life expectancy without deteriorating life quality.

Neuronal sources generate EEG signals with orders of magnitude smaller amplitudes which rules out their contribution to mV-level DC-EEG shifts. Furthermore, subjects in our study were in deep anaesthesia (with suppressed EEG activity at frequencies > 0.5 Hz) that was induced by intravenous thiopental 2 minutes prior to mannitol infusion (Fig 1). It has been shown in animal experiments that deep levels of anaesthesia can disrupt BBB integrity and thereupon generate a shift in scalp DC-EEG [50,51]. Thiopental induced a DC-EEG shift comparable to that seen in animal studies [25]. However, it was of much smaller amplitude than the mannitol response, suggesting that a robust effect on BBB was induced by mannitol only. The subjects in our study were normo-ventilated with stable end-tidal CO2, which excludes the possibility of the known hypo-/hypercapnia related DC shifts generated across an intact BBB [24]. The routine use of intravenous atropine prior to BBBD prevents the known vasovagal changes and their contribution to the DC-EEG signals. Taken together, the DC-EEG shifts seen in our study during the BBBD procedure can be fully accounted for by non-neuronal, non-respiratory yet brain-confined signal sources.

The time window observed in the DC-EEG potential response to mannitol is perfectly in line with the known BBB penetrance time window for large particles (5–200 nm; nanoparticles and viruses) that lasts maximally for 15 minutes following BBB disruption in animal models [16,52]. We demonstrate the potential of spatiotemporal mapping of this time window in humans using topographic maps. Such information may be very important in the development of new therapeutic approaches to currently non-treatable brain diseases that are inaccessible to pharmacotherapy because of an intact BBB. Real-time BBBD monitoring can enable the personnel to optimize induction of BBB permeabilization within a safe therapeutic window and to judge if the subsequent BBB recovery should be augmented by cortisol after the therapy.

As expected, the intravenous thiopental bolus-related DC-potential shift showed no lateralization. In contrast to this, the responses upon mannitol infusion via either left or right carotid artery were strongly lateralized and they indicate differential local effects in the generation of the DC-EEG shift. That the contralateral side showed a response of opposite polarity is at least partly a consequence of using the common average reference montage, but qualitatively similar results were seen with other montages (data not shown). Accordingly, vertebral artery mannitol infusions resulted in responses with a frontal-posterior distribution and little lateralization. These responses were smaller in amplitude, which is consistent with the location of the infused arterial territory and the mechanism that couples BBB-generated signals to scalp [24]. Thus the DC-EEG potential shifts follow arterial territories and enable quantitative mapping of the BBBD with commonly available topographic mapping methods. Taken together, DC-EEG enables real-time spatiotemporal monitoring of BBBD induced by intra-arterial mannitol infusions in PCNSL chemotherapy, and it may prove to be a useful tool in a wide variety of therapeutic interventions in the future.

Changes in brain hemodynamics induced by jugular vein compression, Valsalva or Müller manoeuvres, show good correlation between NIRS vs. DC-EEG shifts generated by the intact BBB [30]. Therefore we used NIRS in the present study to provide further information on the origin of BBBD-associated DC-EEG shifts. The main finding with carotid artery infusions was a marked triphasic cerebrovascular response that began with a rapid fall in Hb and HbO, consistent with dilution of blood during the 30 sec mannitol infusion. As in an animal model [53], this was followed by a hyperaemic second phase (30 sec – 2 min) showing markedly elevated HbO and a transient partial recovery of Hb. Furthermore, the NIRS time course is in line with the increase in cerebral blood flow velocity observed in pigs using transcranial Doppler monitoring immediately after mannitol-induced BBBD [3]. Thereafter within a minute, Hb fell again and HbO showed further increase resulting in levels that only partly recovered whereas the DC-EEG signals largely recovered by the end of the 20 min monitoring period. Notably, the Hb and HbO changes after the early bolus effects were almost linear in contrast to the non-linear DC-EEG changes. The prolonged fall in the Hb level cannot be explained merely by hyperaemia induced dilution, but rather it reflects temporary cessation of oxygen consumption since deoxyhaemoglobin is not being produced. These findings support the conclusion that the DC-EEG changes indeed reflect BBB disruption and its subsequent gradual sealing and not responses of the intact BBB upon changes in brain hemodynamics.

Recently, several diseases have been linked to the disruption of the protective properties of the BBB. Ischemia, degenerative diseases, inflammatory diseases, neoplasms and homeostatic disturbances all compromise the integrity of the BBB [38]. On the other hand, recent advances in focused ultrasound suggest that BBBD can be done less invasively in the near future in humans [54]. This opens new horizons for developing augmented drug delivery in diseases affecting either the BBB itself or the neuroglial tissue behind it [55]. According to our present results, non-invasive DC-EEG could be readily coupled with such new methods for continuous monitoring during treatment. Further development of DC-EEG for controlled drug delivery applications could benefit from the in vivo optical imaging methods that provide means of quantifying BBB penetrance of drugs in animal models [56]. Moreover, our setup is compatible with ultrafast MRI, which could be used to obtain complementary information on the brain status.

## Conclusions

Our results demonstrate the feasibility of DC-EEG for real-time monitoring of induced transient BBBD in anesthetized human patients receiving chemotherapy for PCNSL. In addition to providing valuable real-time information on BBBD during PCNSL treatment, our present results and the DC-EEG method may be exploited when devising novel therapeutic strategies involving BBBD-aided pharmacotherapy of brain diseases.
